# Murine Trophoblast Stem Cells and Their Differentiated Cells Attenuate Zika Virus In Vitro by Reducing Glycosylation of the Viral Envelope Protein

**DOI:** 10.3390/cells10113085

**Published:** 2021-11-09

**Authors:** Biswas Neupane, Mona Fendereski, Farzana Nazneen, Yan-Lin Guo, Fengwei Bai

**Affiliations:** Department of Cell and Molecular Biology, Center for Molecular and Cellular Biosciences, The University of Southern Mississippi, Hattiesburg, MS 39406, USA; Biswas.Neupane@usm.edu (B.N.); Mona.Fendereski@usm.edu (M.F.); Farzana.Nazneen@usm.edu (F.N.); yanlin.guo@usm.edu (Y.-L.G.)

**Keywords:** trophoblast stem cells (TSCs), Zika virus, envelop protein, glycosylation

## Abstract

Zika virus (ZIKV) infection during pregnancy can cause devastating fetal neuropathological abnormalities, including microcephaly. Most studies of ZIKV infection in pregnancy have focused on post-implantation stage embryos. Currently, we have limited knowledge about how a pre-implantation stage embryo deals with a viral infection. This study investigates ZIKV infection on mouse trophoblast stem cells (TSCs) and their in vitro differentiated TSCs (DTSCs), which resemble the cellular components of the trophectoderm layer of the blastocyst that later develops into the placenta. We demonstrate that TSCs and DTSCs are permissive to ZIKV infection; however, ZIKV propagated in TSCs and DTSCs exhibit substantially lower infectivity, as shown in vitro and in a mouse model compared to ZIKV that was generated in Vero cells or mouse embryonic fibroblasts (MEFs). We further show that the low infectivity of ZIKV propagated in TSCs and DTSCs is associated with a reduced level of glycosylation on the viral envelope (E) proteins, which are essential for ZIKV to establish initial attachment by binding to cell surface glycosaminoglycans (GAGs). The decreased level of glycosylation on ZIKV E is, at least, partially due to the low-level expression of a glycosylation-related gene, *Hexa*, in TSCs and DTSCs. Furthermore, this finding is not limited to ZIKV since similar observations have been made as to the chikungunya virus (CHIKV) and West Nile virus (WNV) propagated in TSCs and DTSCs. In conclusion, our results reveal a novel phenomenon suggesting that murine TSCs and their differentiated cells may have adapted a cellular glycosylation system that can limit viral infectivity by altering the glycosylation of viral envelope proteins, therefore serving as a unique, innate anti-viral mechanism in the pre-implantation stage embryo.

## 1. Introduction

Zika virus (ZIKV) is a mosquito-borne *Flavivirus* belonging to the family *Flaviviridae* [1]. Other important human pathogenic viruses belonging to the same family include the dengue virus (DENV), the West Nile virus (WNV), the yellow fever virus (YFV), the tick-borne encephalitis virus (TBEV), and the Japanese encephalitis virus (JEV) [2]. ZIKV is transmitted through day-biting mosquitoes, *Aedes* *aegypti* and *Aedes albopictus.* ZIKV was first isolated in the Zika forest of Uganda in 1947. For a few decades, its infection in humans only caused mild symptoms, including fever, headache, myalgia, arthralgia, rash, and conjunctivitis, until the major outbreak in Brazil in 2015 [3]. During that outbreak, many ZIKV infection cases were linked to severe neurological diseases that include intrauterine growth restriction, congenital microcephaly, head growth deceleration in infants [4,5,6], and Guillain-Barré syndrome (GBS) in adults [7].

The flaviviruses consist of a single-stranded, positive-sense RNA genome, which encodes a polyprotein that is cleaved into three structural (capsid, pre-membrane, and envelope) and seven non-structural proteins (NS1, NS2a, NS2b, NS3, NS4a, NS4b, and NS5) [2,8]. The flavivirus envelope (E) protein is a transmembrane glycoprotein that mediates cell surface receptor binding and endocytosis [2]. ZIKV and other flaviviruses make initial contact with the host cell through E protein binding to glycosaminoglycans (GAGs), such as heparan-sulfate proteoglycans or syndecans. GAGs are highly sulfated polysaccharides expressed on the cell surface and extracellular matrix of mammalian cells [9,10]. They are prominently exposed on the cell surfaces of all tissues, providing an easily accessible primary receptor for viral adhesion by electrostatic interactions [8]. It has been reported that DENV, YFV, JEV, TBEV, Murray encephalitis virus, WNV, and chikungunya virus (CHIKV) can use GAG receptors for the initial host cell attachment [11,12,13,14]. In addition, the interaction of ZIKV E protein with different GAGs has also been reported [15], and the level of glycosylation on E protein affects ZIKV attachment and infectivity [15,16]. Viral glycoproteins are glycosylated via post-translational modifications in the host cell. It has been reported that viruses propagated in different host cells may compose different glycosylation patterns on their glycoproteins, thus affecting virus–host attachment and replication [17]. 

Trophoblasts are the first differentiated lineage that mediates blastocyst implantation to the uterine epithelium [18]. TSCs are multipotent stem cells and primarily reside in the polar trophectoderm region of the blastocyst that can differentiate into specialized sub-types of trophoblasts for placenta development [19]. TSCs can be induced to differentiate into trophoblasts in vitro; therefore, they have been used as a model to study placental development [2]. The placenta provides protection and nutrients to the developing embryo; however, its immune protective function to the fetus is not well-recognized [20]. Some viruses, including ZIKV, can cross the blood–placenta barrier, infect the fetus, and cause devastating neuropathological abnormalities [21]. However, whether or to what extent the trophectoderm in a blastocyst can offer immune protection is not clear. In this study, we investigate ZIKV infection of mouse TSCs and their in vitro differentiated TSCs (DTSCs). Our results reveal a novel phenomenon suggesting that murine TSCs and their differentiated cells may have adapted a cellular glycosylation system that can limit viral infectivity by altering the glycosylation of viral envelope proteins, therefore serving as a unique, innate anti-viral mechanism in the pre-implantation stage embryo.

## 2. Methods and Materials

### 2.1. Ethics Statement and Biosafety

All animal care and experiments were conducted according to the Guide for the Care and Use of Laboratory Animals under protocol #16031002, which was approved by the Institutional Animal Care and Use Committee (IACUC) of The University of Southern Mississippi (USM). All the experiments involving live ZIKV, CHIKV, and WNV were performed by certified personnel in biosafety level 2 and 3 laboratories, following standard biosafety protocols approved by the USM Institutional Biosafety Committee.

### 2.2. Viruses, Cells, and Animals 

ZIKV (strain PRVABC59) was obtained from Dr. B. Johnson (CDC Arbovirus Branch, Fort Collins, CO, USA), and CHIKV (LR OPY1 2006 strain) was provided by the University of Texas Medical Branch. WNV (CT2741) was provided by Dr. John F. Anderson at the Connecticut Agricultural Experiment Station. All viruses were propagated in Vero cells (ATCC CCL-81), and the viral stocks were titrated in Vero cells by plaque assay as described previously [22].

### 2.3. Cells

Vero cells were cultured in Dulbecco’s modified Eagle’s medium (DMEM, Life Technologies, Grand Island, NY, USA) containing 1% l-glutamine, 1% penicillin/streptomycin, and 10% fetal bovine serum (FBS). Mouse TSCs were provided by Dr. Wei Hsu (University of Rochester Medical Center) and cultured in RPMI 1640 containing 20% FBS, 1 mM sodium pyruvate, 2 mM l-glutamine, 50 units/mL penicillin, 50 μg/mL streptomycin, 100 μM 2-mercaptoethanol, 25 ng/mL FGF4, and 1 μg/mL heparin, with 70% of the medium pre-conditioned on mouse embryonic fibroblasts (MEFs). All cells were maintained at 37 °C in a humidified incubator with 5% CO_2_. For the differentiation of TSCs, TSCs (50–60% confluence) were cultured in unconditioned TSC basal medium without FGF4 and heparin for 5–6 days. The differentiated cells, designated as DTSCs, were used for further studies under the conditions specified in individual experiments. For the generation of MEFs, male and female C57BL/6J mice were paired, and the appearance of the vaginal plug was monitored the following morning. The presence of the vaginal plug was considered as embryonic day 0.5 (E0.5). On E14.5, the pregnant female mice were sacrificed for the collection and generation of MEFs, as previously described [23]. 

### 2.4. Mice

Breeding pairs of type I interferon receptor-deficient (*Ifnar1^−/−^*) mice with a C57BL/6J background were purchased from the Jackson Laboratory (Bar Harbor, ME, USA). The breeders and the pups were housed under standard conditions in a clean room, and viral infection studies were carried out in the BSL-3 animal facility at USM. Four-week-old *Ifnar1^−/−^* mice were weighed and infected with 1 × 10^4^ PFU of ZIKV generated in different cells in 50 μL of PBS containing 1% FBS via footpad. Blood samples were collected on days 2, 4, and 6 p.i. to measure the copy numbers of *ZIKV* by qPCR, and the viral burden was expressed as log_10_ of PFU equivalent per ml of blood. Mice were observed daily for survival up to day 30 p.i.

### 2.5. Attachment Assay

Cells were plated in 12-well plates at a density of 2 × 10^5^ cells per well, incubated at 37 °C with 5% CO_2_ for 24 h. The cells were then infected with 1 MOI of different types of ZIKV in cold (4 °C) growth medium, and the plates were kept at 4 °C for 1 h. After incubation, the wells were washed with cold PBS three times to remove the unattached viruses, and the cells were collected in TRIreagent (Molecular Research Center, Inc., Cincinnati, OH, USA) for total RNA isolation and qPCR quantification of *ZIKV*.

### 2.6. Plaque Assay

Vero cells were plated in 6-well plates at a density of 6 × 10^5^ cells per well, incubated at 37 °C with 5% CO_2_ for 24 h. Virus-containing media were added to the wells and incubated at 37 °C with 5% CO_2_ for 1 h. After incubation, the unattached virus was removed, and the wells were covered with the first overlay medium and incubated at 37 °C with 5% CO_2_ until the observation of plaques. The plaques were stained with Neutral Red, present in the second overlay medium, then counted [22].

### 2.7. Quantitative PCR (qPCR)

Cells or mouse blood were collected for total RNA extraction with TRIreagent and converted into first-strand cDNA using the iSCRIPT^TM^ cDNA synthesis kit (Bio-Rad, Hercules, CA, USA). Probe-based (Bio-Rad) qPCR was performed using iTAQ^TM^ polymerase supermix for the detection of *ZIKV*, *CHIKV-E1*, *WNV E*, and cellular *β-actin*, as described previously [14,24,25,26,27]. For the relative quantification of *Hexa* (Forward: CGTCGCTGAGAGACTGTGGAG, Reverse: CCAGCTCACAACGGAAATGCG), SYBR Green-based (Bio-Rad) qPCR was performed and normalized to cellular *β-actin*.

### 2.8. Heparin Sepharose Bead Binding Assay

Heparin-conjugated sepharose beads or heparin-unconjugated beads (60 µL) were washed with DMEM, and 1 × 10^5^ PFU of ZIKV in a volume of 60 µL was added to the beads. The mixture was incubated at 4 °C for 30 min to let the viruses attach to the beads. The unattached viruses were removed by washing the beads three times with DMEM containing 2% FBS. The bound viruses were lysed in 50 µL of Laemmli sample buffer (Alfa Aesar, Haverhill, MA, USA), and the proteins were separated by SDS-polyacrylamide gel electrophoresis. The proteins were then transferred to a nitrocellulose membrane (Bio-Rad) and blocked for 1 h with 5% bovine serum albumin at RT. Mouse monoclonal anti-flavivirus primary antibody (4G2) was used to probe the membranes at 4 °C overnight on a rocker. After washing the membrane with Tris-buffered saline with Tween 20 (TBS-T), horseradish peroxidase-conjugated goat anti-mouse IgG secondary antibody (Jackson Immunoresearch, West Grove, PA, USA) was added for 1 h at RT. The membrane was washed with TBS-T and developed using SuperSignal West Pico Chemiluminescent Substrate (Thermo Scientific, Rockford, IL, USA) for acquiring the images using a ChemiDoc MP system (Bio-Rad). 

### 2.9. Concentrating of ZIKV and Protein Glycosylation Assay

The culture media of ZIKV_Vero_, ZIKV_TSC_, and ZIKV_DTSC_ was centrifuged at 5000 rpm for 5 min, and the supernatants were UV-inactivated. The viral particles were collected after ultracentrifugation through 20% sucrose with 28,000 rpm at 4 °C for 2 h (Beckman Coulter, Brea, CA, USA). The viral proteins were de-glycosylated using peptide-N-glycosidase F (PNGase F, Sigma, St. Louis, MO, USA) following the manufacturer’s instructions. ZIKV E protein was probed with the 4G2 antibody in an immunoblotting assay, as above.

### 2.10. Immunoblotting Assay for HEXA

Vero cells transfected with *Hexa* siRNA were infected with 1 MOI of ZIKV. After 24 h, the cells were collected and lysed in Laemmli sample buffer (Alfa Aesar, Haverhill, MA, USA). The proteins were then separated by 10% SDS-polyacrylamide gel electrophoresis and transferred to a nitrocellulose membrane (Bio-Rad) and blocked for 1 h with 5% bovine serum albumin at RT. After treating with mouse-specific Rabbit primary antibody (HEXA, Abcam, Boston, MA, USA) in the ratio 1:1000 at 4 °C overnight on a rocker, the membrane was washed with TBS-T. Horseradish peroxidase-conjugated secondary antibody (Goat polyclonal Ab to Rabbit IgG, Abcam; 1:5000) was added for 1 h at RT; the membrane was washed with TBS-T and developed using SuperSignal West Pico Chemiluminiscence Substrate (Thermo Scientific, Waltham, MA, USA) for acquiring the images using a ChemiDoc MP system (Bio-Rad). 

### 2.11. qPCR Array

MEFs and TSCs were plated in 12-well plates at a concentration of 2 × 10^5^ cells per well. After 24 h, the cells were collected, and total RNA was extracted with TRIreagent (Molecular Research Center, Inc., Cincinnati, OH, USA) and converted into the first-strand cDNA using an iSCRIPT^TM^ cDNA synthesis kit (Bio-Rad). RT^2^ Profile PCR Array (Qiagen, Germantown, MD, USA) was performed in a 96-well plate treated with SYBR Green-optimized primer assays for mouse glycosylation-related genes following the manufacturer’s protocol. 

### 2.12. Statistical Analyses

Data analysis was performed using Student’s *t*-test or analysis of variance (ANOVA) in GraphPad Prism software (version 6.0), *p* < 0.05 being considered statistically significant.

## 3. Results

### 3.1. ZIKV Propagated in TSCs and DTSCs Exhibits Reduced Infectivity In Vitro

**Figure 1:**
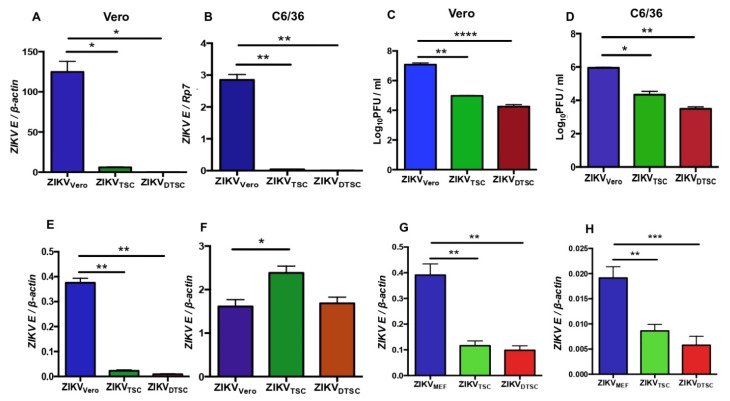
ZIKV generated in TSCs and DTSCs decreases infectivity. Vero cells and C6/36 were infected with ZIKV (MOI = 1) that was propagated in Vero cells (ZIKV_Vero_), TSCs (ZIKV_TSC_), or DTSCs (ZIKV_DTSC_) for 24 h. The ratio of *ZIKV* to Vero *β-actin* (**A**) or C6/36 *Rp7* (**B**) was determined by qPCR. ZIKV titers in the cell media were determined by plaque assay (**C**, Vero and **D**, C6/36). (**E**) Vero cells were inoculated with 1 MOI of ZIKV_Vero_, ZIKV_TSC_, or ZIKV_DTSC_ and incubated at 4 °C for 1 h. After washes, the attached viruses were quantified with qPCR. (**F**) Replication of ZIKV_TSC_ and ZIKV_DTSC_ after passaging one generation on Vero cells. Vero cells were infected with 1 MOI of the Vero-generated ZIKV_TSC_ or ZIKV_DTSC_ for 24 h. The ratio of *ZIKV* to Vero *β-actin* was determined by qPCR. (**G**,**H**) Mouse embryonic fibroblasts (MEFs) were used as control cells to generate ZIKV_MEF_. (**G**) ZIKV (1 MOI) grown in MEFs, TSCs, and DTSCs were used to infect Vero cells for 24 h, and the levels of viral replication were quantified by qPCR by measuring ZIKV, normalized to cellular *β-actin*. (**H**) Vero cells were inoculated with ZIKV grown in different cells for 1 h at 4 °C, and the attached viruses were quantified by qPCR. The data are representative of at least two independent experiments and expressed as mean ± the standard errors of the mean (SEM). The results were analyzed by using a two-tailed Student’s *t*-test. *, **, ***, and **** denote *p* < 0.05, *p* < 0.01, *p* < 0.001, and *p* < 0.0001, respectively.

**Figure 2:**
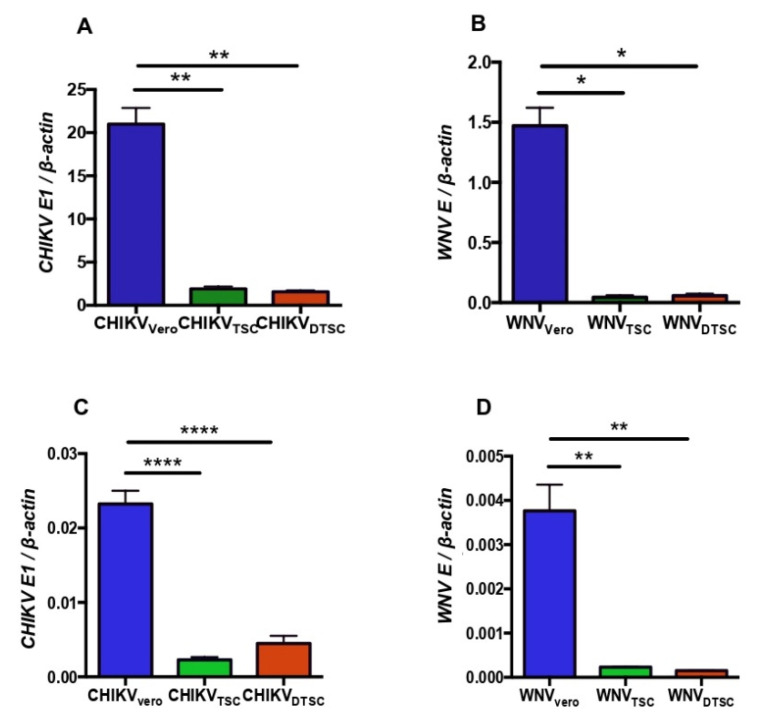
CHIKV and WNV generated in TSCs, and DTSCs decrease infectivity in vitro. Vero cells were infected with 1 MOI of CHIKV (**A**) or WNV (**B**) that was generated in Vero, TSCs, or DTSCs. At 24 h p.i., the ratio of *CHIKV-E1* or *WNV E* to cellular *β-actin* was determined by qPCR. Vero cells were inoculated with 1 MOI of CHIKV (**C**) and WNV (**D**) grown in the different cells and incubated at 4 °C for 1 h. After washes, the attached viruses were quantified with qPCR. The results were analyzed by using a two-tailed Student’s *t*-test and are representative of at least two independent experiments and expressed as mean ± SEM. *, **, and **** denote *p* < 0.05, *p* < 0.01, and *p* < 0.0001, respectively.

To test the infectivity of ZIKV in TSCs and DTSCs, we infected TSCs and DTSCs with ZIKV and collected the cell media; ZIKVs propagated in these cells were named ZIKV_TSC_ and ZIKV_DTSC_, respectively. For comparative analysis, Vero cells were also infected with ZIKV under the same condition, and the produced viruses were named ZIKV_Vero_. ZIKV_Vero_ developed plaques between 4–6 days in a plaque-forming assay, whereas it took 7–10 days for ZIKV_TSC_ and ZIKV_DTSC_ to form plaques, indicating weak infectivity of the viruses derived from TSCs and DTSCs. The infectivity of these viruses was further examined by infecting Vero cells with a reverse-transcription quantitative PCR (qPCR) assay. The results showed that the replication of ZIKV_TSC_ and ZIKV_DTSC_ was approximately 20-fold and 100-fold lower than ZIKV_Vero_ at 24 h post-infection (p.i.), respectively (Figure 1A). As ZIKV is primarily transmitted by mosquitoes, we also assessed the infectivity of ZIKV_TSC_ and ZIKV_DTSC_ in mosquito C6/36 cells. Similarly, we found that the infectivity of ZIKV_TSC_ and ZIKV_DTSC_ was also significantly decreased compared to ZIKV_Vero_ in C6/36 cells (Figure 1B). To confirm these qPCR results, we performed a plaque-forming assay to measure the viral titers in the cell culture media. Consistently, the results showed that ZIKV_TSC_ and ZIKV_DTSC_ decreased by approximately 100-fold the infectious viral particles in both Vero and C6/36 cell cultures (Figure 1C,D). To test if the reduced infectivity of ZIKV_TSC_ and ZIKV_DTSC_ may be due to reduced attachment to the host cells, we incubated Vero cells with 1 MOI of ZIKV propagated in different cells at 4 °C for 1 h. At this temperature, viruses can attach to the cells but cannot enter inside. After incubation, the cells were washed with cold PBS to remove unbounded viruses, and the attached viruses were quantified by qPCR. In comparison to ZIKV_Vero_, the attachment of ZIKV_TSC_ and ZIKV_DTSC_ was significantly lower (Figure 1E). Interestingly, ZIKV_TSC_ and ZIKV_DTSC_ can regain their lost infectivity after re-propagation in Vero cells for one additional passage (Figure 1F). These results suggest that ZIKV generated in TSCs and DTSCs has lower attachment and infectivity, which may result from the different enzymatic machinery in the host cells. To exclude the possibility that this phenomenon is due to the origin of the Vero cells (African Green Monkey), we compared the infectivity of ZIKV_TSC_ and ZIKV_DTSC_ with ZIKV generated in mouse embryonic fibroblasts (MEFs). The results of viral RNA replication and the attachment assay also indicated that ZIKV-propagated TSCs and DTSCs displayed reduced viral replication (Figure 1G) and attachment (Figure 1H). We next tested if this phenomenon is only limited to ZIKV. For this, we propagated WNV (another flavivirus) and CHIKV (an alphavirus) in TSCs, DTSCs, or Vero cells. Similarly, our results demonstrated that both CHIKV (Figure 2A) and WNV (Figure 2B) propagated in TSCs and DTSCs exhibited attenuated replication (Figure 2A,B) and attachment to the host cells (Figure 2C,D) when compared to the viruses generated in Vero cells. These results collectively demonstrate that ZIKV, WNV, and CHIKV propagated in TSCs and DTSCs exhibit reduced infectivity in vitro, suggesting that an intrinsic deficiency in these stem cells and their differentiated cells may attenuate these viruses.

### 3.2. ZIKV_TSC_ and ZIKV_DTSC_ Exhibit Attenuated Infectivity in Ifnar1^−/−^ Mice

**Figure 3:**
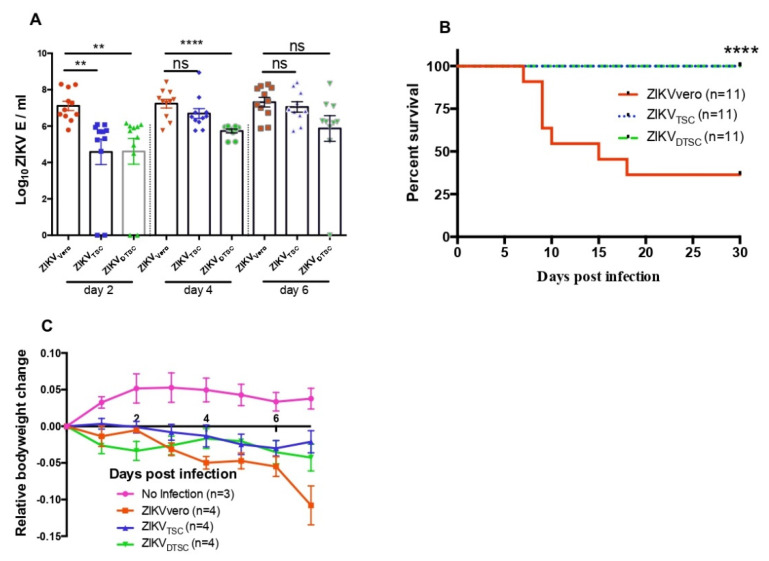
ZIKV_TSC_ and ZIKV_DTSC_ exhibit attenuated infectivity in *Ifnar1^−/−^* mice. Four-week-old *Ifnar1^−/−^* mice were infected with 1 × 10^4^ PFU of ZIKV_Vero_, ZIKV_TSC_, or ZIKV_DTSC_ via footpad. (**A**) The ZIKV genome copies were quantified by measuring *ZIKV* with qPCR and expressed as log_10_ (PFU/mL). (**B**) The survival curves of *Ifnar1^−/−^* mice infected with the different strains of ZIKV. (**C**) Relative bodyweight changes of the mice compared to the weight before infection (Day 0). The data were analyzed by a two-tailed Student *t*-test (**A**) or log-rank test (**B**). ** and **** denote *p* < 0.01, and *p* < 0.0001, respectively.

To evaluate the infectivity of ZIKV_TSC_ and ZIKV_DTSC_ in a mouse model, we infected 4-week-old type I Interferon receptor-deficient (*Ifnar1^−/−^*) mice with 1 × 10^4^ PFU of ZIKV generated in different cells through footpad inoculation. Blood was collected on days (D) 2, 4, and 6 p.i., and the *ZIKV E* level was compared. Consistent with the in vitro results, the *ZIKV E* levels in the blood of the mice inoculated with ZIKV_TSC_ and ZIKV_DTSC_ were significantly lower than those infected with ZIKV_Vero_ on D2 p.i. With a similar trend, the levels of viremia slowly increased in ZIKV_TSC_ and ZIKV_DTSC_-infected mice at later time points (Figure 3A). These ZIKV-infected mice were monitored daily for 30 days, and the survival analysis shows that 35% of ZIKV_Vero_-infected mice versus 100% of ZIKV_TSC_- and ZIKV_DTSC_-infected mice survived (Figure 3B). In addition, we measured the weight loss of the mice for 7 days before the mice started dying and found a trend that ZIKV_Vero_-infected mice lost more bodyweight than the mice infected with ZIKV_TSC_ or ZIKV_DTSC_ (Figure 3C). These in vivo results indicate that ZIKV_TSC_ and ZIKV_DTSC_ have attenuated infectivity in mice.

### 3.3. ZIKV_TSC_ and ZIKV_DTSC_ Have Reduced Glycosylation on E Proteins

**Figure 4:**
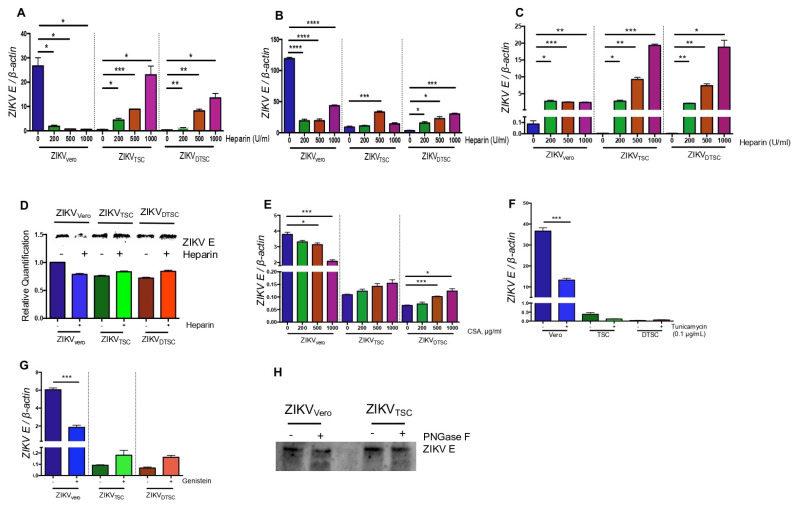
ZIKV grown in TSCs and DTSCs has reduced glycosylation. (**A**) Vero cells were pre-treated with heparin at different concentrations for 1 h, then 1 MOI of ZIKV_Vero_, ZIKV_TSC_, or ZIKV_DTSC,_ and incubated at 4 °C for 1 h. After washes, the attached viruses were quantified with qPCR. (**B**) Vero cells were pre-treated with heparin at different concentrations for 1 h, then inoculated with 1 MOI of ZIKV_Vero_, ZIKV_TSC_, or ZIKV_DTSC_ and incubated at 37 °C for 24 h, and the ratio of *ZIKV* to cellular *β-actin* was determined by qPCR. (**C**) ZIKV_Vero_, ZIKV_TSC_, or ZIKV_DTSC_ were pre-incubated with heparin at 37 °C for 1 h. Then, the mixture of the virus and heparin was added to Vero cell culture (1 MOI) and kept at 4 °C for 1 h, and the cell-attached viruses were quantified by qPCR. (**D**) ZIKV_Vero_, ZIKV_TSC_, and ZIKV_DTSC_ (1 × 10^5^ PFU) were mixed with heparin-conjugated sepharose beads or unconjugated sepharose beads as control and kept at 4 °C for 1 h. After washing, viruses bound to the beads were lysed and analyzed for ZIKV E by an immunoblotting assay (upper panel). The relative quantification of the blots is expressed in bar graphs in the lower panel. (**E**) Vero cells were pre-treated with different concentrations of chondroitin sulfate A (CSA) for 1 h and incubated with 1 MOI of ZIKV_Vero_, ZIKV_TSC_, or ZIKV_DTSC_ at 4 °C for 1 h. After washes, the attached viruses were quantified with qPCR. (**F**) Vero cells, TSCs, and DTSCs were infected with ZIKV (1 MOI) in the presence or absence of tunicamycin. On 24 h p.i., the viral replication was quantified by measuring the ratio of *ZIKV E* to cellular *β-actin* with qPCR. (**G**) Vero cells were incubated with 1 MOI of ZIKV_Vero_, ZIKV_TSC_, or ZIKV_DTSC_ in the presence or absence of genistein at 4 °C for 1 h. After washes, the attached viruses were quantified with qPCR. (**H**) The stocks of ZIKV_Vero_, ZIKV_TSC_, and ZIKV_DTSC_ were pelleted by ultracentrifugation. The viruses were treated with PNGase F for deglycosylation and then analyzed with immunoblotting for ZIKV. The results were analyzed by either one-way ANOVA followed by Tukey’s test (**A**–**C**,**E**) or a two-tailed Student’s *t*-test (**F**,**G**). The results are representative of at least two independent experiments and are expressed as mean ± SEM. *, **, ***, and **** denote *p* < 0.05, *p* < 0.01, *p* < 0.001, and *p* < 0.0001, respectively.

Like other flaviviruses, the attachment of ZIKV to the host cell receptors is mediated by E protein. Although different cell surface receptors have been reported for ZIKV and other flaviviruses, glycosaminoglycan (GAG) receptors play essential roles in flavivirus infectivity. The binding of E protein to GAG receptors is the initial step in the attachment of viruses to the cell surface, and the levels of glycosylation of E by the host cellular machinery have been shown to affect the virus–host cell binding [28]. We thus hypothesized that the lower binding ability of ZIKV_TSC_ and ZIKV_DTSC_ might be due to the deficiency in glycosylation of TSCs and DTSCs. To test this, we pre-incubated Vero cells with different concentrations of a soluble GAG, heparin, at 37 °C for 1 h, then inoculated them with ZIKV generated in TSCs and DTSCs (MOI = 1) at 4 °C for 1 h for attachment analysis by qPCR. The results showed that the pre-treatment of heparin reduced the attachment of ZIKV_Vero_ to the cells in a concentration-dependent manner, whereas the attachment of ZIKV_TSC_ and ZIKV_DTSC_ was promoted by heparin (Figure 4A). Consistent with the binding results, the pre-treatment of Vero cells with heparin inhibited the replication of ZIKV_Vero_ but promoted the replication of ZIKV_TSC_ and ZIKV_DTSC_ (Figure 4B). Interestingly, the pre-treatment of ZIKV dramatically increased the binding of ZIKV_TSC_ and ZIKV_DTSC_ to Vero cells in a heparin-concentration-dependent manner. In contrast, the pre-treatment of ZIKV_Vero_ can also promote viral binding, which, however, did not further increase with heparin at concentrations above 200 U/mL (Figure 4C). To confirm these results, equal PFUs of ZIKV grown in different cells were incubated with heparin-conjugated sepharose beads or unconjugated control beads at 4 °C for 30 min. After washing the unbound viruses, the beads were collected for immunoblotting by probing the ZIKV E protein. Similar to the qPCR results, we found that heparin inhibited the binding of ZIKV_Vero_, but supported the binding of ZIKV_TSC_ and ZIKV_DTSC_ (Figure 4D). To test if other GAGs have the same effect as heparin, we tested the viral binding after the pre-treatment of Vero cells with chondroitin sulfate A (CSA). Like heparin, the CSA pre-treatment also inhibited the binding of ZIKV_Vero_ but showed a trend to support the attachment of ZIKV_TSC_ and ZIKV_DTSC_ (Figure 4E). These results suggest ZIKV_TSC_ and ZIKV_DTSC_ have a lower level of or incomplete glycosylation in their E proteins, which may be due to a deficiency in the glycosylation machinery of TSCs and DTSCs. 

To test this hypothesis, we treated Vero cells with two antibiotics that have been shown to interrupt the process of cellular glycosylation. The first one is tunicamycin, which inhibits the formation of N-acetylglucosamine-lipid intermediates and prevents the glycosylation of newly synthesized glycoproteins [29]. We tested if the presence of tunicamycin in cell media inhibits ZIKV replication in the host cells. For this, we infected Vero cells, TSCs, and DTSCs for 24 h in the presence of tunicamycin (0.1 µg/mL) or DMSO as a negative control. The qPCR results showed that Tunicamycin treatment significantly inhibited ZIKV replication in Vero cells but not in TSCs and DTSCs (Figure 4F). Another antibiotic is genistein, which inhibits the synthesis of GAGs in mammalian cells [30]. To test if the presence of genistein inhibits the attachment of ZIKV to the host cells, we pre-treated Vero cells with genistein for 4 h and inoculated them with 1 MOI of ZIKV_TSC_, ZIKV_DTSC_, or ZIKV_Vero_ for the viral binding analysis by qPCR. The binding results showed that genistein inhibited the attachment of ZIKV_Vero_, but not ZIKV_TSC_ and ZIKV_DTSC_ (Figure 4G). To further confirm the hypothesis that the ZIKV particles generated in TSCs and DTSCs have reduced glycosylation in their E proteins, we treated ZIKV_TSC_ particles with PNGase F, an enzyme that cleaves N-linked glycan from glycoproteins [31], and examined digested E protein fragments by immunoblotting. In ZIKV E, one conserved N-glycosylation site has been reported at N^154^ [32]. As expected, we observed that a relatively smaller band was produced in the ZIKV_Vero_ sample, but not in the ZIKV_TSC_ sample, in the presence of PNGase F, confirming our hypothesis that ZIKV_TSC_ may have reduced glycosylation in their E proteins (Figure 4H). 

### 3.4. The Expression of Hexa Was Decreased in TSCs and DTSCs

**Figure 5:**
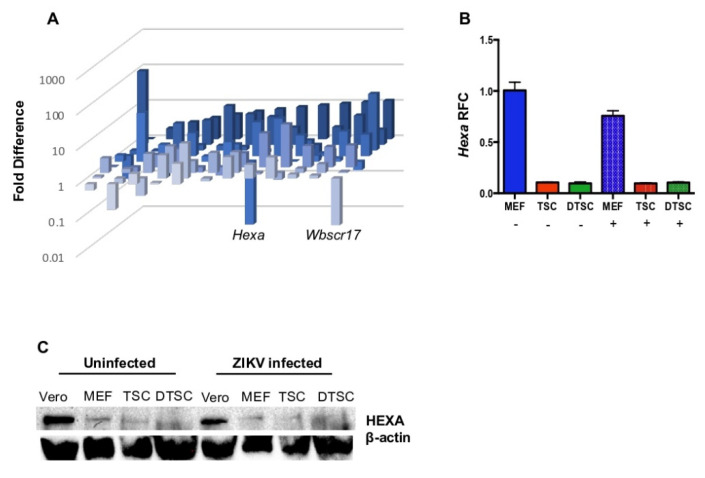
The expression of the glycosylation-related gene and *Hexa* was decreased in TSCs and DTSCs. (**A**) qPCR array analysis of mouse glycosylation-related genes in MEFs and TSCs. (**B**) MEFs, TSCs, and DTSCs were infected with ZIKV (1 MOI) for 24 h, and the expression of *Hexa* was measured and related to the uninfected MEFs and normalized with cellular *β-actin*. (**C**) The reduced level of HEXA in TSCs and DTSCs was further confirmed at the protein level. The levels of HEXA and β-Actin in ZIKV-infected and uninfected Vero, MEFs, TSCs, and DTSCs were quantified by immunoblotting assay. The results were analyzed by using a two-tailed Student’s *t*-test (**B**). The results are representative of at least two independent experiments and are expressed as mean ± SEM (**B**).

To pin down which gene may be related to the deficiency in glycosylation in TSCs and DTSCs, we screened the expression of mouse glycosylation-related genes with a qPCR array. The array analysis showed that *Hexa* expression was downregulated 58-fold in TSCs compared to the MEF control cells (Figure 5A). To confirm this result, we infected the MEFs, TSCs, and DTSCs with ZIKV (1 MOI) or PBS as control and analyzed *Hexa* expression by qPCR. The results confirmed that the expression of *Hexa* in TSCs and DTSCs decreased 44- and 55-fold, respectively, compared to that in MEF cells at 24 h of infection (Figure 5B). In addition, there was the same trend of reduction of HEXA in TSCs and DTSCs as in Vero and MEF cells at the protein level in an immunoblotting analysis (Figure 5C). These results indicate that HEXA may contribute to the glycosylation of viral glycoproteins when the viruses replicate in the host cells.

### 3.5. HEXA Contributes to the Glycosylation of ZIKV in TSCs and DTSCs

**Figure 6:**
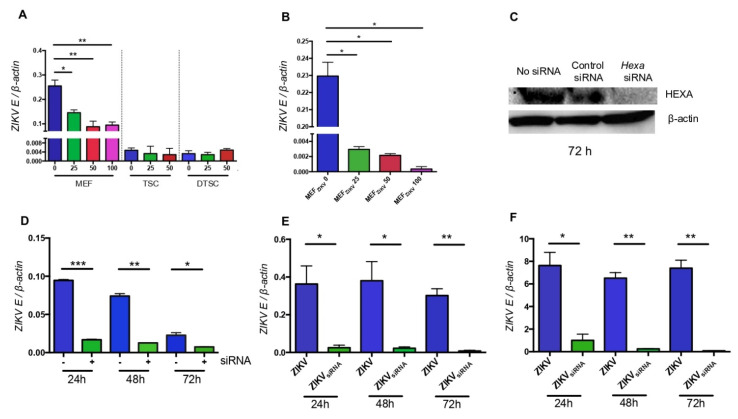
HEXA contributes to the glycosylation of ZIKV during the replication in TSCs and DTSCs. (**A**) MEFs, TSCs, and DTSCs were pre-treated with Z-Pugnac at different concentrations for 4 h, then infected with ZIKV (1 MOI) for 24 h, and the viruses in the cells were quantified by qPCR. (**B**) Vero cells were infected with ZIKV generated in MEFs in the presence of Z-Pugnac for 24 h, and the viral replication was quantified by measuring the ratio of *ZIKV E* to cellular *β-actin* with qPCR. (**C**) Vero cells were transfected with *HexA* siRNA or randomized siRNA as a control for 72 h. The levels of HEXA and β-Actin were measured by immunoblotting. (**D**) Vero cells were transfected with *HexA* siRNA, then infected with ZIKV (1 MOI). After 24 h of infection, the level of *ZIKV E* was quantified by qPCR and normalized to *β-actin*. (**E**) ZIKV and ZIKV grown in *HexA* siRNA-transfected cells were used to infect Vero cells with 0.1 MOI. After 1 h of infection at 4 °C, the level of viruses attached to the cells was quantified by qPCR. (**F**), Vero cells were infected with 0.1 MOI of ZIKV and ZIKV grown in the presence of *HexA* siRNA for 24, 48, and 72 h at 37 °C for 24 h, and the levels of the viral replication were measured by qPCR. The results were analyzed by either one-way ANOVA followed by Tukey’s test (**A**,**B**) or a two-tailed Student’s *t*-test (**D**–**F**). The results are representative of at least two independent experiments and are expressed as mean ± SEM. *, **, and *** denote *p* < 0.05, *p* < 0.01, and *p* < 0.001, respectively.

HEXA is an O-linked N-acetyl-D-glucosaminidase (OGA), which plays a vital role in protein glycosylation. UDP-O-linked-N-acetyl glucosamine (UDP-GlcNAc) produced from the hexosamine biosynthetic pathway (HBP) is used for glycosylation with the help of OGT (O-GlcNAc transferase), whereas it is removed and added back to the pathway by OGA for the recycling of UDP-GlcNAc. Thus, a balance between the levels of OGT and OGA in the cells is required for proper glycosylation. It has already been reported that an unbalanced level of HEXA can impair glycosylation [33]. After finding the possible role of HEXA in ZIKV E glycosylation, we asked if the inhibition of HEXA in the host cells reduces ZIKV infectivity. To test this, we first treated MEFs, TSCs, and DTSCs with a HEXA inhibitor, Z-Pugnac, and evaluated if the inhibition of HEXA reduces ZIKV replication. The results showed that after treating the cells with Z-Pugnac, ZIKV replication was inhibited in MEFs but not in TSCs and DTSCs (Figure 6A), suggesting that HEXA contributes to the replication of ZIKV in MEF cells. In addition, we infected Vero cells with 1 MOI of the ZIKV released from Z-Pugnac-treated MEF cell media and quantified *ZIKV* by qPCR. Similar to ZIKV_TSC_ and ZIKV_DTSC_, ZIKV generated in Z-Pugnac-treated MEFs exhibited attenuated infectivity compared with the control, i.e., ZIKV propagated in MEFs in the absence of the inhibitor (Figure 6B). We next tested if knocking down the expression of *Hexa* using siRNA would also show the same phenotype. After confirming that the expression of *Hexa* was successfully knocked down by the siRNA (Figure 6C), we transfected Vero cells with *Hexa* siRNA and then inoculated them with ZIKV. The qPCR analysis shows that the knockdown of *Hexa* inhibits ZIKV replication (Figure 6D). We then examined the attachment (Figure 6E) and replication of ZIKV in Vero cells (Figure 6F). Both results suggest that ZIKV propagated in *Hexa* siRNA-treated Vero cells (ZIKV_siRNA_) exhibits reduced attachment and infectivity. In summary, the inhibition and siRNA knockdown results demonstrate that host cell HEXA plays an essential role in supporting ZIKV binding and replication, possibly through enhancing E protein glycosylation. 

## 4. Discussion

An early embryo is the most crucial stage in the life cycle of mammals, and it faces dynamic immunological challenges during embryogenesis [20]. The blastocyst consists of two major components: the inner cell mass (ICM) and the trophectoderm, which give rise to the fetus and the placenta, respectively. A series of our recent studies have demonstrated that embryonic stem cells (ESCs) derived from the ICM have an underdeveloped interferon-meditated anti-viral system [34]. This is a surprising finding since the IFN response is a critical innate anti-viral immunity, presumably developed in most, if not all, cell types in vertebrate animals [35]. Increasing evidence suggests that early embryos may have adapted distinct anti-viral mechanisms that are different from a developed organism [34]. As part of our effort to characterize the immune properties of the early embryonic cells, this study used multiple experimental approaches and demonstrated that the trophectoderm may utilize glycosylation as a unique anti-viral strategy to protect the early embryo from viral infection. 

Congenital ZIKV infection has been associated with neuronal birth defects in newborns [36]. As ZIKV can cross the placenta and infect the fetus, it is plausible to hypothesize that ZIKV may first infect TSCs and DTSCs, then the fetus. Interestingly, our results demonstrate that ZIKV generated in TSCs and DTSCs exhibit significantly lower infectivity in Vero cells, C6/36 cells, and mice. However, after passing a single passage of ZIKV_TSC_ and ZIKV_DTSC_ on Vero cells, these viruses regained the lost infectivity, indicating some intrinsic deficiencies related to post-translational modifications in TSCs and DTSCs (compared with Vero cells) that mitigate ZIKV infectivity. In addition, this phenomenon remained true when we compared the infectivity of ZIKV generated in TSCs and DTSCs with those generated in MEFs. Further, the attenuation of infectivity was also noticed in CHIKV and WNV after growing in TSCs and DTSCs.

The first step of a viral life cycle is the attachment to the host cell. Although a few molecules have been shown to mediate ZIKV E binding to the host cells, a specific cellular receptor for ZIKV is still not known [37]. Mammalian cell surface GAG receptors have been reported to play a significant role in the initial attachment for different viruses [15,38,39,40]. Our previous study in CHIKV has shown that CHIKV propagated in C6/36 cells exhibited a lower GAG-binding ability than those grown in Vero cells [14]. The interaction of ZIKV E protein with different GAGs has also been reported [15], and the level of glycosylation on E protein affects ZIKV attachment and infectivity [16]. Similarly, our GAG receptor neutralization results with heparin showed that ZIKV_TSC_ and ZIKV_DTSC_ have reduced binding to GAG receptors. 

Viral proteins are glycosylated via post-translational modifications in the host cell. It has been reported that viruses propagated in different host cells may compose different glycosylation patterns on their glycoproteins, thus affecting virus–host attachment and replication [17,41]. Enveloped viruses can acquire a portion of the host cell membrane as their envelope via budding, which can cause differences in the carbohydrate and lipid composition of the virus [42,43,44]. Further, different cell types use different enzymes for the post-translational modification process during N-glycosylation to modify the viral glycoproteins [45,46]. In addition, the carbohydrate residues at the glycosylation sites also depend on the type of cells used to propagate the viruses. In this study, we have demonstrated that ZIKV_TSC_ has a reduced level of glycosylation on E proteins by treatment with PNGase F. The deficiency in glycosylation of ZIKV_TSC_ and ZIKV_DTSC_ was also confirmed with the experiments of propagating ZIKV in Vero cells in the presence of Tunicamycin or Genistein, both of which can inhibit the process of glycosylation of mammalian cells [30,47]. The presence of either Tunicamycin or Genistein in the cell culture of Vero cells significantly reduces the infectivity and attachment of ZIKV_Vero_, but not of ZIKV_TSC_ or ZIKV_DTSC_. Besides E protein, PrM/M is another glycoprotein of flaviviruses. A recent study showed that removing the N-glycosylation site from the prM or both prM and E in a ZIKV infectious clone did not result in infectious ZIKV [48]. The absence of the N-glycan on prM or E led to protein aggregation in the rough endoplasmic reticulum (ER) compartment, which was more pronounced when N-glycan on prM was removed [48]. Although we did not assess the level of glycosylation on the PrM/M of ZIKV_TSC_ or ZIKV_DTSC_, it is possible that attenuated infectivity is also contributed by the incomplete glycosylation of PrM/M, which needs further investigation. 

Glycosylation is an inducible and reversible post-translational modification of proteins [49]. A set of enzymes belonging to O-GlcNAc transferase (OGT) can transfer the GlcNAc residue from UDP-GlcNAc to the serine or threonine residues of the target proteins [50,51]. Similarly, another set of O-GlcNAcase (OGA) enzymes can remove GlcNAc from the proteins [52,53]. The level of glycosylation is sensitive to the nutrients in the cellular environment, and the differences in the nutrient status, hormone levels, and extracellular environmental stress can change the level of GlcNAc in the proteins [54,55]. The level of the OGT and OGA has been reported to be altered in stem cells [56,57,58]. In this study, we found that the expression of one of the members of OGA, *Hexa*, is significantly reduced in TSCs and DTSCs compared to MEFs and Vero cells. As OGA helps recycle GlcNAc to the HBP pool through the salvage pathway [59], the decreased expression of *Hexa* can disturb the glycosylation process. Further, studies have reported that the expression of OGT and OGA is sensitive to fluctuations at cellular GlcNAc levels, and cells can coordinate their expression to buffer themselves from drastic shifts in glycosylation [60,61,62,63]. Further, the mutation in *Hexa* has also been linked with a congenital disorder in humans, Tay Sachs disease, in which the growth and development of the brain is inhibited [64]. In this study, we inhibited the expression of *Hexa* in Vero cells by using Z-Pugnac or siRNA. In both cases, ZIKV generated in *Hexa*-inhibited conditions decreased its infectivity, suggesting that *Hexa* at least partially contributes to the glycosylation of ZIKV E protein, which is deficient in mouse TSCs and DTSCs. It is worth noting that the expression of another member of OGT (*Wbscr17*) was also downregulated in TSCs, albeit to a lesser extent compared to *Hexa* (Figure 5A). It is possible that the reduced expression of *Wbscr17* may also contribute to the attenuation of ZIKV in stem cells, which needs further investigation.

**Figure 7:**
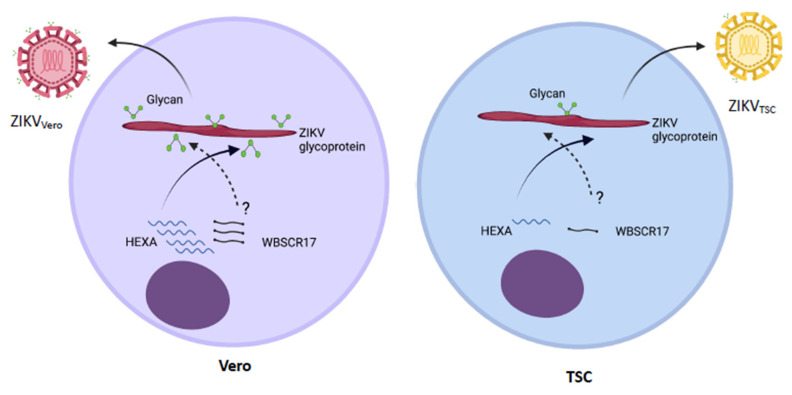
Schematic illustration of the mechanism of HEXA-mediated glycosylation of ZIKV proteins. Vero cells produce HEXA and possibly WBSCR17 during ZIKV replication, which facilitate the glycosylation of ZIKV E and other glycoproteins. In contrast, TSCs and DTSCs have a reduced level of HEXA during ZIKV replication, resulting in less glycosylation of ZIKV E proteins, which attenuates ZIKV binding to GAG receptors. Created with Biorender.com.

In conclusion, this study has discovered a novel phenomenon that viruses generated in TSCs and DTSCs, including ZIKV, WNV, and CHIKV, exhibit attenuated infectivity in cultured cells and reduced pathogenicity in mice. Mechanistically, we have demonstrated that this is partly due to the unique glycosylation system in TSCs and DTSCs that produces a low level of viral protein glycosylation, thus limiting virus attachment to and invasion of host cells, as shown in Figure 7. Physiologically, we propose that this could be a unique, innate anti-viral mechanism adapted by early embryos.

## Data Availability

The data presented in this study are available on request from the corresponding author.
